# Anterior Nares Diversity and Pathobionts Represent Sinus Microbiome in Chronic Rhinosinusitis

**DOI:** 10.1128/mSphere.00532-19

**Published:** 2019-11-27

**Authors:** Ilke De Boeck, Stijn Wittouck, Katleen Martens, Jos Claes, Mark Jorissen, Brecht Steelant, Marianne F. L. van den Broek, Sven F. Seys, Peter W. Hellings, Olivier M. Vanderveken, Sarah Lebeer

**Affiliations:** a University of Antwerp, Department of Bioscience Engineering, Antwerp, Belgium; b KU Leuven, Department of Microbiology and Immunology, Research Group Allergy and Clinical Immunology, Leuven, Belgium; c Clinical Department of Otorhinolaryngology, Head and Neck Surgery, University Hospitals Leuven, Leuven, Belgium; d University of Antwerp, Faculty of Medicine and Health Sciences, Translational Neurosciences, Antwerp, Belgium; e Antwerp University Hospital, ENT, Head and Neck Surgery, Edegem, Belgium; University of Michigan—Ann Arbor

**Keywords:** microbiome, upper respiratory tract, chronic rhinosinusitis, sinus pathobionts

## Abstract

It is generally believed that the microbiome plays a role in the pathophysiology of chronic rhinosinusitis (CRS), though its exact contribution to disease development and severity remains unclear. Here, samples were collected from the anterior nares, nasopharynx, and maxillary and ethmoid sinuses of 190 CRS patients and from the anterior nares and nasopharynx of 100 controls. Microbial communities were analyzed by Illumina sequencing of the V4 region of 16S rRNA. The phenotype and patient characteristics were documented, and several serum inflammatory markers were measured. Our data indicate a rather strong continuity for the microbiome in the different upper respiratory tract (URT) niches in CRS patients, with the microbiome in the anterior nares being most similar to the sinus microbiome. Bacterial diversity was reduced in CRS patients without nasal polyps compared to that in the controls but not in CRS patients with nasal polyps. Statistically significant differences in the presence/absence or relative abundance of several taxa were found between the CRS patients and the healthy controls. Of these, Dolosigranulum pigrum was clearly more associated with URT samples from healthy subjects, while the Corynebacterium tuberculostearicum, Haemophilus influenzae/H. aegyptius, and *Staphylococcus* taxa were found to be potential pathobionts in CRS patients. However, CRS versus health as a predictor explained only 1 to 2% of the variance in the microbiome profiles in an adonis model. A history of functional endoscopic sinus surgery, age, and sex also showed a minor association. This study thus indicates that functional studies on the potential beneficial versus pathogenic activity of the different indicator taxa found here are needed to further understand the pathology of CRS and its different phenotypes. (This study has been registered at ClinicalTrials.gov under identifier NCT02933983.)

**IMPORTANCE** There is a clear need to better understand the pathology and specific microbiome features in chronic rhinosinusitis patients, but little is known about the bacterial topography and continuity between the different niches of the upper respiratory tract. Our work showed that the anterior nares could be an important reservoir for potential sinus pathobionts. This has implications for the diagnosis, prevention, and treatment of CRS. In addition, we found a potential pathogenic role for the Corynebacterium tuberculostearicum, Haemophilus influenzae/H. aegyptius, and *Staphylococcus* taxa and a potential beneficial role for *Dolosigranulum*. Finally, a decreased microbiome diversity was observed in patients with chronic rhinosinusitis without nasal polyps compared to that in healthy controls but not in chronic rhinosinusitis patients with nasal polyps. This suggests a potential role for the microbiome in disease development or progression of mainly this phenotype.

## Abstract (precis)

There is a clear need to better understand the pathology and specific microbiome features in chronic rhinosinusitis patients, but little is known about the bacterial topography and continuity between the different niches of the upper respiratory tract. Our work showed that the anterior nares could be an important reservoir for potential sinus pathobionts. This has implications for the diagnosis, prevention, and treatment of CRS. In addition, we found a potential pathogenic role for the Corynebacterium tuberculostearicum, Haemophilus influenzae/H. aegyptius, and *Staphylococcus* taxa and a potential beneficial role for *Dolosigranulum*. Finally, a decreased microbiome diversity was observed in patients with chronic rhinosinusitis without nasal polyps compared to that in healthy controls but not in chronic rhinosinusitis patients with nasal polyps. This suggests a potential role for the microbiome in disease development or progression of mainly this phenotype.

## INTRODUCTION

Chronic rhinosinusitis (CRS) is a chronic inflammatory disorder, characterized by inflammation of the sinonasal cavity, with symptoms lasting more than 12 weeks (1). The disease has a prevalence of 11% and 13.4% in Europe and the United States, respectively (2, 3). Despite available treatment and evidence-based guidelines, CRS remains uncontrolled in up to 40% of patients even after sinus surgery (1, 4). Therefore, there is the need to better understand the pathology of CRS, which may require differentiation of patient subgroups. Generally, two major phenotypes are distinguished: CRS with nasal polyps (CRSwNP) and CRS without nasal polyps (CRSsNP) (1). However, subclassifying CRS into CRSwNP and CRSsNP does not provide a full insight into the underlying pathophysiology (5, 6). We suggest that specific microbiome features could provide a further differentiation of patient subgroups and benefit the development of new therapeutic strategies (3, 6, 7).

Recently, studies revealed a possible role for the microbiome in the pathology of CRS, based on altered bacterial diversity and the involvement of certain pathogenic bacteria (8–14), but the results of the different studies are conflicting. More specifically, some studies reported a decreased bacterial alpha diversity in CRS patient samples compared to that in samples from controls (8, 10), whereas others did not find significant differences in alpha diversity (11, 12). Several research groups have also tried to identify bacterial taxa that could have a potential pathogenic role in aggravating CRS or a beneficial function in preventing or reducing the risk for CRS. For instance, Corynebacterium tuberculostearicum/C. accolens and Staphylococcus aureus appear to be significantly enriched in the sinuses or middle meatus (i.e., a nasal passage of the nasal cavity, located between the middle meatus turbinate and lateral nasal wall) of CRS patients (8, 14–17). On the contrary, other microorganisms, such as *Lactobacillus* (8), *Propionibacterium* (recently reclassified to *Cutibacterium* [18]), *Burkholderia* (with which a meta-analysis was performed [19]), and *Peptoniphilus* (11, 20), have been suggested to be taxa that might promote sinus health. Nevertheless, much remains to be discovered about the topographical occurrence and function of potential pathobionts and beneficial microorganisms in specific locations of the upper respiratory tract (URT) in CRS and other chronic airway diseases.

In this study, we performed an integrated analysis of the topographical differences and continuity of four sites of the URT, i.e., the anterior nasal cavity, nasopharynx, and maxillary and ethmoid sinuses, of CRS patients. We investigated whether the microbiomes of the anterior nasal cavity and/or nasopharynx were representative of the maxillary and ethmoid sinus microbiomes. Therefore, samples from 225 CRS patients were collected, subjected to Illumina MiSeq sequencing, and analyzed at a fine-scale, amplicon sequence variant (ASV) level. Samples from CRS patients (*n* = 225) and healthy subjects (*n* = 100) were compared to explore differences in bacterial alpha and beta diversity and to identify taxa that might have an impact on disease severity and/or health. Finally, the microbiome profiles were correlated with patient characteristics (i.e., age, sex, medical treatment, smoking behavior, and disease severity), CRS phenotype features (i.e., nasal polyps, allergies, and asthma), and the concentrations of inflammatory markers (i.e., interleukin-5 [IL-5], IL-13, IL-4, and interferon gamma [IFN-γ]) measured in the serum of the CRS patients.

## RESULTS

### Microbiome continuity in different URT sites of CRS patients.

CRS patients (*n* = 225) were recruited during functional endoscopic sinus surgery (FESS), and their anterior nares, nasopharynx, and maxillary and ethmoid sinuses were sampled. For each site, 82%, 80%, 77%, and 78% of the samples, respectively, passed the quality pipeline, which was carefully implemented because of the low-biomass nature of these URT niches. As such, 190 CRS patients with samples from at least one URT site with a high-quality profile were included (Table 1).

**TABLE 1:** Characteristics of CRS patients^*a*^

Characteristic	Value for:	
	Patients with CRS (*n* = 190)	Control participants (*n* = 100)
Mean ± SD age (yr)	42 ± 13	34 ± 11
% of patients with the following characteristics:		
Male	63	39
Nonsmoker	61	85
Allergy	32	16
Asthma	22	0
Polyposis	44	NA
Prior surgery (FESS)	43	NA
Nasal and/or oral steroids	85	NA
Preoperative antibiotics	41	NA
Purulence	31	NA
Mean ± SD SNOT-22 score	51 ± 19	NA
Mean ± SD VAS (total symptom score)	6.8 ± 2.2	NA
Geometric mean concn (pg/ml)		
Periostin	46.4 ± 51.4	NA
IFN-γ	14.8 ± 31	NA
IL-5	0.7 ± 0.7	NA
IL-4	Below detection limit	NA
IL-13	Below detection limit	NA

a Age, sex, the 22-item Sino-Nasal Outcome Test (SNOT-22) score, the Visual Analog Scale (VAS) score, medical treatment (nasal/oral steroids and antibiotics in last 3 months), smoking behavior, and a history of FESS were recorded via a questionnaire and, if available, checked in the patient’s medical record. SNOT-22 and VAS are widely validated scoring systems whose scores reflect the severity of the disease. Both scoring systems are evaluated by use of a list of disease-related symptoms (58, 59). Also, phenotypic characteristics of self-reported asthma, allergies (based on total IgE and the results of skin prick tests for allergies to common inhalant allergens), and nasal polyps were documented. Lastly, the concentrations of different inflammatory cytokines (periostin, interleukin-4 [IL-4], IL-13, IL-5, and interferon gamma [IFN-γ]) in serum samples were determined. IL-5, IL-4, and IL-13 are important regulators of type 2 inflammation in CRSwNP patients, and IFN-γ is involved in non-type 2 inflammation in CRSsNP patients. Additionally, periostin has been postulated to be a potential diagnostic marker for asthma and is involved in many aspects of allergic inflammation, including the development of a Th2 immune response. NA, not available.

**FIG 1:**
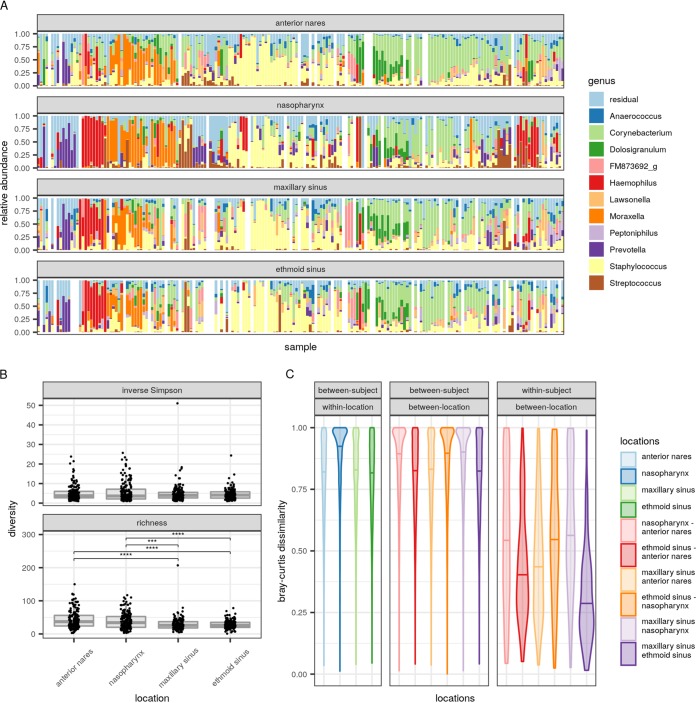
Bacterial profiles and diversity of the different URT sites sampled in CRS patients. (A) Dominant genera in the anterior nares, nasopharynx, and maxillary and ethmoid sinus samples. The order of the samples is determined by hierarchical clustering on pooled samples per participant. (B) Comparison of the inverse Simpson index (top) and richness (bottom) of the different URT sites sampled in CRS patients at the ASV level. *P* values (determined by unpaired Welch *t* tests with the Holm-Bonferroni correction for multiple testing) of less than 0.05 were considered significant. Asterisks represent statistically significant differences between the niches. ***, *P* ≤ 0.001; ****, *P* ≤ 0.0001. (C) Bray-Curtis dissimilarities as an indicator of intrapersonal and interpersonal differences between the nose, nasopharynx, and maxillary and ethmoid sinuses at the ASV level; horizontal bars represent median dissimilarity values.

*Staphylococcus*, *Corynebacterium*, and *Moraxella* were the most prevalent genera across all URT sites (Fig. 1A) (samples were pooled per participant, and hierarchical clustering was performed on these pooled samples), with mean relative abundances of 22%, 21%, and 7.2%, respectively. Although the four sites showed high similarity in the bacterial genera that dominated the samples, sampling site explained a small but statistically significant proportion of the microbial variation (2.2%; *P* ≤ 0.001, adonis test). Certain nasopharynx samples showed a more divergent bacterial profile, with the samples being enriched with *Haemophilus*, *Streptococcus*, and *Prevotella* (Fig. 1A; see also Fig. S1 in the supplemental material). The last two taxa were more abundant in the nasopharynx than in the anterior nares and sinuses. For both taxa, relative abundances were 8% in the nasopharynx, while they were less than 4% in the other niches (Table S1). *Haemophilus* dominated a subset of the maxillary and ethmoid sinus samples, with mean relative abundances of 5% and 6%, respectively; in comparison, the abundances were 11% in the nasopharynx and only 2% in the anterior nares (Table S1).

10.1128/mSphere.00532-19.2 FIG S1 Principal-coordinate analysis (PCoA) to compare bacterial taxonomy and relative abundances between samples at the same location and interindividual variation between locations within CRS patients, based on Bray-Curtis dissimilarity. Download FIG S1, TIF file, 0.5 MB. Copyright © 2019 De Boeck et al. 2019 De Boeck et al. This content is distributed under the terms of the Creative Commons Attribution 4.0 International license.

10.1128/mSphere.00532-19.6 TABLE S1 Mean relative abundances. Mean relative abundances of the taxa that were overall the most abundant in the various sites sampled in CRS patients. N, anterior nares; NF, nasopharynx; SE, ethmoid sinus; SM, maxillary sinus. Download Table S1, CSV file, 0.002 MB. Copyright © 2019 De Boeck et al. 2019 De Boeck et al. This content is distributed under the terms of the Creative Commons Attribution 4.0 International license.

In the next step, alpha diversity (richness and the inverse Simpson index) was calculated (Fig. 1B) at the level of ASVs. The average inverse Simpson indices and richness were low, highlighting that only a limited number of bacterial ASVs dominated the anterior nares, nasopharynx, and maxillary and ethmoid sinuses of a given person. Inverse Simpson indices (Fig. 1B, top) were not significantly different among the four sites. In contrast, richness was significantly different, with the highest taxon richness being seen in the anterior nares (*P* ≤ 0.0001 compared to both sinuses, *t* test) and the nasopharynx (*P* ≤ 0.001 compared to the maxillary sinus and *P* ≤ 0.0001 compared to the ethmoid sinus) (Fig. 1B, bottom). The richness between the anterior nares and nasopharynx and the richness between the maxillary and ethmoid sinus were not statistically significantly different (*P* > 0.05).

To further explore the bacterial topography and continuity of the different URT sites at the inter- and intrapersonal levels, Bray-Curtis dissimilarities between the microbiomes at different sites in the same participant (Fig. 1C, right) and in different participants (Fig. 1C, left and middle) were calculated. Within the same participant, the microbiome structures of the maxillary and ethmoid sinuses were most similar to each other, with a median Bray-Curtis dissimilarity of 0.27. For the anterior nares, median dissimilarities of 0.43 (maxillary sinus) and 0.40 (ethmoid sinus) were observed. For the nasopharynx, these median dissimilarities with the maxillary and ethmoid sinuses were 0.58 and 0.56, respectively. Bray-Curtis dissimilarities between samples from different participants were generally high (median > 0.80 for sample pairs both from the same site and from different sites), indicating that the continuity between the different URT sites is an intrapersonal feature (Fig. 1C, left and middle). Together, we observed a rather high continuity between the different URT sites within participants, where the microbiome of the anterior nares even showed a slightly higher similarity with the sinus microbiome than with the nasopharynx microbiome, based on Bray-Curtis dissimilarity.

### Bacterial diversity in the anterior nares and nasopharynx is lower in CRSsNP patients.

Since a continuity between the microbial community of both the anterior nares and the nasopharynx with that of the sinuses was observed in CRS patients, we next evaluated samples from both sites for a detailed comparison of the microbiomes between healthy controls and CRS patients. For ethical reasons, only samples from the anterior nares and nasopharynx were collected from healthy controls. Within the patient group, 174 samples from the anterior nares and 172 samples from the nasopharynx with high-quality profiles were obtained. For the control group, these numbers were 86 and 94, respectively.

**FIG 2:**
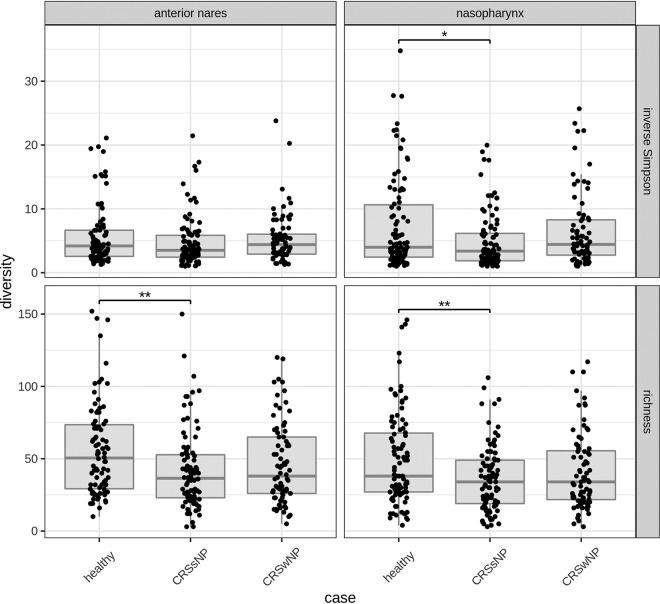
Comparison of alpha diversity measures in the anterior nares (left) and nasopharynx (right) between healthy controls, CRSsNP patients, and CRSwNP patients. Asterisks represent statistically significant differences between the niches (determined by unpaired Welch *t* tests with the Holm-Bonferroni correction for multiple testing). *, *P* ≤ 0.05; **, *P* ≤ 0.01.

To compare the microbiomes of the anterior nares and nasopharynx of our CRS patients with those of the healthy controls, alpha diversity was measured (richness and inverse Simpson index) (Fig. 2). Our patient group was divided into those with CRSsNP and those with CRSwNP, since CRS is generally characterized by two major clinical phenotypes, depending on the presence or absence of nasal polyps. Overall richness was significantly lower in the anterior nares (*P* ≤ 0.01, *t* test) and nasopharynx in patients with CRSsNP than in the controls, and also, the inverse Simpson index was significantly decreased in the nasopharynx of patients with CRSsNP (*P* ≤ 0.05) compared to the controls. Alpha diversity was not decreased in patients with CRSwNP compared to the controls. Overall, our data indicate decreased bacterial alpha diversity in CRSsNP patients compared to the healthy controls.

### Specific bacterial taxa are enriched or diminished in CRS patients.

**FIG 3:**
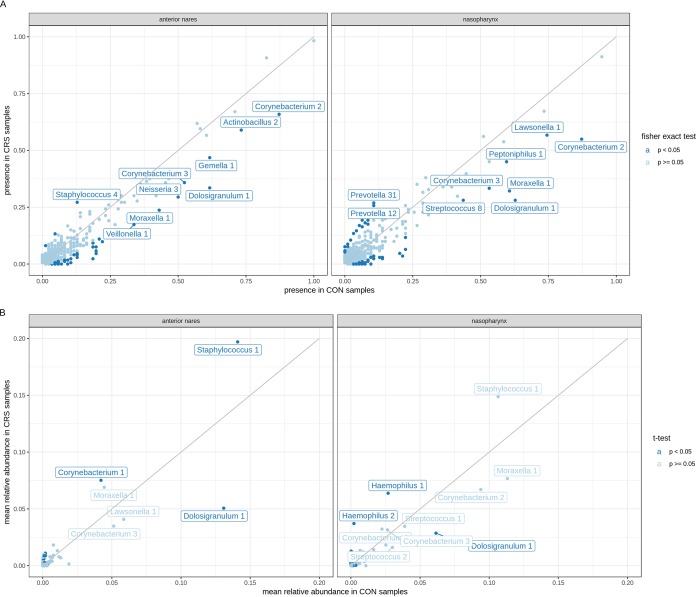
Differences in the presence/absence and relative abundance of the most prevalent taxa in CRS patients versus healthy controls (CON). (A) Correlation between the presence of ASVs in healthy controls and CRS patients in the anterior nares (left) and the nasopharynx (right). A Fisher exact test was used to test for the significance of ASVs that were more present in healthy controls or CRS patients (*P* ≤ 0.05). Only ASVs with a significant presence and more than 25% presence under at least one of the conditions are shown with a name label. (B) Correlation between the mean relative abundance of ASVs in the anterior nares (left) and the nasopharynx (right) of healthy controls and CRS patients. Only ASVs with a mean relative abundance of greater than 30% under at least one of the conditions are shown with a name label.

To explore specific microbiome differences between healthy controls and CRS patients, the effect size of disease status in the study population was analyzed. For the anterior nares, only 2% of the variation observed within the bacterial community composition could be explained by whether a participant was healthy or had CRS (*P* ≤ 0.001, adonis test). For the nasopharynx, this variation was 1% (*P* ≤ 0.01). Next, the bacterial profiles between healthy controls and CRS patients were compared at the level of the presence or absence of ASVs, as well as their relative abundances in the anterior nares and nasopharynx (Fig. 3 and Table S2). Only ASVs with a presence of more than 25% under at least one of the conditions and with a significant difference between both conditions are shown with a name label in Fig. 3A. ASVs with a mean relative abundance greater than 0.03 under at least one of the conditions are shown with a label in Fig. 3B. In the anterior nares, *Staphylococcus* ASV 2 (*Staphylococcus* 2) was significantly more prevalent in CRS patients than in the controls (26% of the CRS patients versus 13% of the controls) (Fig. 3A, left). Additionally, *Staphylococcus* 1 (mean relative abundance, 0.14 in the controls and 0.20 in CRS patients) and *Corynebacterium* 2 (mean relative abundance, 0.04 in the controls and 0.08 in CRS patients) were relatively more abundant in CRS patients than in the controls (Fig. 3B, left). Comparison of the sequences of the ASVs with the sequences in the EzBioCloud 16S rRNA database (21) showed that these ASVs likely belong to the species Staphylococcus aureus/S. argenteus/S. capitis/S. caprae/S. epidermidis/S. haemolyticus and Corynebacterium tuberculostearicum, although other closely related species are also possible. *Moraxella* 1, *Corynebacterium* 1 and 3, *Dolosigranulum* 1, *Streptococcus* 5, *Actinomyces* 1, and *Neisseria* 3 were more prevalent in healthy controls. However, only *Dolosigranulum* 1 (likely Dolosigranulum pigrum, on the basis of the database used and because no other species of this bacterial genus are currently identified) was significantly more associated with the anterior nares of healthy controls than with those of CRS patients, based on presence or absence and relative abundance (present in 62% of control subject samples and 30% of CRS patient samples; mean relative abundance, 0.13 in control subjects and 0.05 in CRS patients).

10.1128/mSphere.00532-19.7 TABLE S2 Overview of the different metadata collected from the CRS patients. Download Table S2, CSV file, 0.01 MB. Copyright © 2019 De Boeck et al. 2019 De Boeck et al. This content is distributed under the terms of the Creative Commons Attribution 4.0 International license.

In the nasopharynx, *Moraxella* 1, *Corynebacterium* 1 and 3, *Dolosigranulum* 1, and *Neisseria* 3 occurred more in healthy controls than in CRS patients (Fig. 3A, right). *Streptococcus* 5, *Lawsonella* 1, and *Peptoniphilus* 1 were also more present in the nasopharynx of healthy controls than in that of CRS patients. As was the case for the anterior nares, Dolosigranulum pigrum was more prevalent in the nasopharynx of the healthy controls than in that of CRS patients (62% of the healthy controls versus 25% of the CRS patients) and showed a higher relative abundance in the healthy controls than in CRS patients (mean relative abundance, 0.06 in healthy controls versus 0.03 in CRS patients). Two *Haemophilus* ASVs (whose V4 sequences were identical to the V4 sequences of Haemophilus influenzae and Haemophilus aegyptius in our reference database) had a significantly higher relative abundance but not presence in CRS patients compared to the healthy controls. Lastly, one *Prevotella* ASV (*Prevotella* 13, whose sequence was identical to a V4 sequence of Prevotella salivae) was more prevalent in the nasopharynx of CRS patients than in that of the healthy controls (11% of control samples and 25% of CRS patient samples). In conclusion, we found some ASVs belonging to *Staphylococcus*, *Haemophilus*, *Corynebacterium*, and *Prevotella* taxa to be more associated with CRS patients than with healthy controls, based on their presence and/or relative abundance. Based on their presence alone, several ASVs seemed to be more associated with the healthy control group; however, only the *Dolosigranulum* ASV was significantly more associated with the healthy control group than with the CRS patient group, based on its presence and abundance.

### Disease-related characteristics are not associated with microbiome profiles.

Since CRS is characterized by different pheno- and endotypes (3, 6, 7), we studied the microbiome in relation to various relevant features describing phenotypes (i.e., asthma, allergy, polyps, and infection) and inflammatory markers (i.e., periostin, interleukin-4 [IL-4], IL-13, IL-5, and interferon gamma [IFN-γ]). The inflammatory markers were chosen based on literature demonstrating that the cytokines IL-5, IL-4, and IL-13 are important regulators of type 2 inflammation in patients with CRSwNP and that IFN-γ is involved in non-type 2 inflammation, mostly seen in patients with CRSsNP (3). Additionally, periostin has been postulated to be a potential diagnostic marker for asthma and is involved in many aspects of allergic inflammation, including the development of a Th2 immune response (22). Associations were investigated for the nasopharynx samples in the CRS group that passed quality control (*n* = 172).

**FIG 4:**
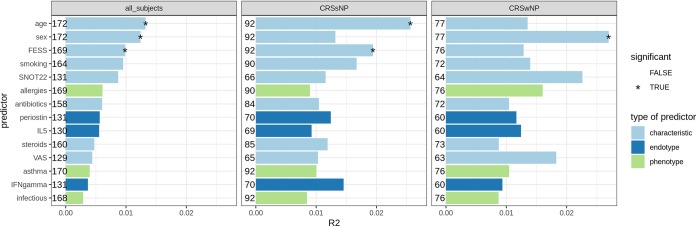
Associations between the nasopharyngeal microbiome profiles of CRS patients (*n* = 172) and covariates. Adonis tests were performed for each covariate for either all CRS subjects (left), only the CRSsNP subjects (middle), or only the CRSwNP subjects (right). The bars represent the effect sizes of the covariates (*R*^2^ values); statistical significance (*P* < 0.05) is indicated with an asterisk. Covariates are colored based on the metadata category. The numbers depicted next to each bar represent the number of subjects used in the adonis model.

First, we tested whether the microbiome was associated with the presence of polyps; this proved not to be the case (*P* = 0.13, adonis test; see Fig. S2). Next, we tested associations between the microbiome and the other metadata variables at the level of the entire CRS patient cohort as well as separately for the CRSsNP and the CRSwNP groups (Fig. 4; Table S2). Minor associations with age, sex, and a history of FESS were found. For the patient characteristics, the microbiome composition in CRS patients was significantly influenced by the age of the patients (*P* = 0.002). This significant effect was seen only in the group of CRSsNP patients. Also, sex showed an association with the overall microbiome structure in the study cohort (*P* = 0.005), but this was significant only in the CRSwNP group. A history of FESS was associated with the overall microbiome only in CRSsNP patients (*P* = 0.043), indicating that these participants who underwent a previous FESS have bacteria slightly different from those in participants who had never had a FESS before. Asthma, allergy, and infection did not show significant associations with the overall microbiome structure, nor did the different inflammatory markers tested (Fig. 4).

**FIG 5:**
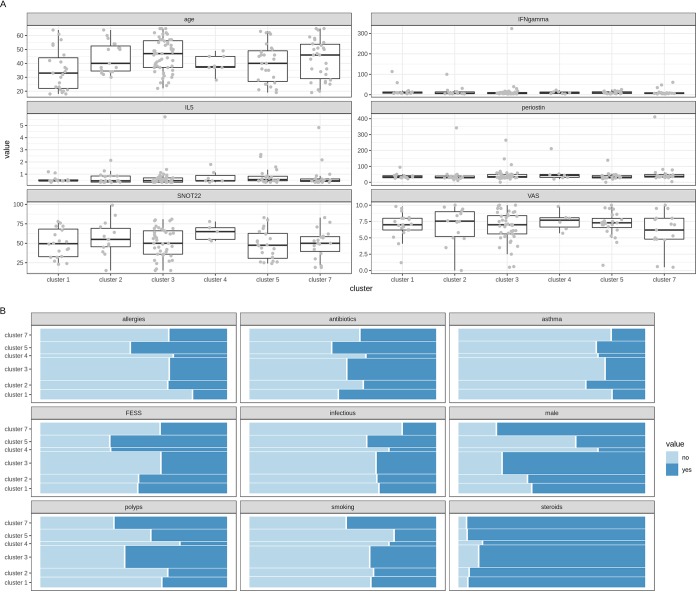
Associations of numerical (A) and categorical (B) microbiome covariates with microbiome-based subject clusters. (A) Box plot visualization of age, IFN-γ concentration, IL-5 concentration, periostin concentration, and 22-item Sino-Nasal Outcome Test (SNOT-22) and Visual Analog Scale (VAS) scores for the six microbiome clusters. (B) Mosaic plot showing the association of the categorical variables with the microbiome clusters. The surface of each colored area is proportional to the number of subjects that it represents. Significance tests of associations of the covariates with the microbiome were performed using the adonis model.

To look deeper into the associations observed for sex, age, and a history of FESS, all patients were clustered into 14 microbiome clusters, based on the abundance of ASVs (Fig. S3). Six clusters (each having more than five participants) were used for further analysis. These were dominated by *Haemophilus* (cluster 1), *Moraxella* (cluster 2), a mixed cluster of *Corynebacterium* and *Staphylococcus* (cluster 3), *Streptococcus* (cluster 4), *Staphylococcus* (cluster 5), and *Prevotella* (cluster 7) (Fig. S3). Cluster 6 and clusters 8 to 14 were not included because they each had less than five participants. We then visually compared these microbiome clusters with all patient variables. Each cluster was analyzed against the numerical (Fig. 5A) and categorical (Fig. 5B) microbiome covariates that were documented. For sex and a history of FESS, female participants and participants with a history of FESS were slightly more present in *Streptococcus* cluster 4 and *Staphylococcus* cluster 5 (Fig. 5B). For age, participants within the *Haemophilus* cluster seemed to have a lower mean age (Fig. 5A). Disease-related parameters, such as polyps, the 22-item Sino-Nasal Outcome Test (SNOT-22) score, and the Visual Analog Scale (VAS) score, showed no association with the clusters, nor did any one of the tested inflammatory markers (Fig. 5). Taken together, our data demonstrate small associations between age, sex, and a history of FESS with the overall microbiome in CRS.

10.1128/mSphere.00532-19.3 FIG S2 Differences in the presence/absence and relative abundance of the most prevalent taxa in CRSsNP versus CRSwNP patients. (A) Correlation between the presence of ASVs in CRS patients without nasal polyps (CRSsNP) and CRS patients with nasal polyps (CRSwNP) in the anterior nares (left) and the nasopharynx (right). A Fisher exact test was used to test for the significance of ASVs that were more present in either patient group (*P* ≤ 0.05). Only ASVs with a significant presence and more than 25% presence under at least one of the conditions are shown with a name label. (B) Correlation between the mean relative abundance of ASVs in the anterior nares (left) and the nasopharynx (right) of CRSsNP and CRSwNP patients. Only ASVs with a mean relative abundance of greater than 30% under at least one of the conditions are shown with a name label. Download FIG S2, TIF file, 0.9 MB. Copyright © 2019 De Boeck et al. 2019 De Boeck et al. This content is distributed under the terms of the Creative Commons Attribution 4.0 International license.

## DISCUSSION

**FIG 6:**
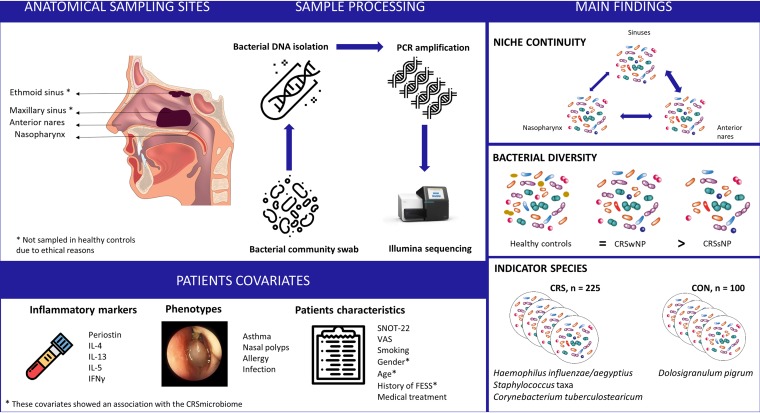
Graphical summary of the URT sampling sites and patient covariates, sample processing, and main findings of this study.

Several studies have explored the URT microbiome in CRS patients but have presented contradictory results regarding the microbiome composition and diversity (as reviewed in reference 23). In this study, the URT microbiome of 225 CRS patients was compared with the microbiome of 100 healthy individuals (24). We analyzed the similarities of the microbiomes in the anterior nares, nasopharynx, and the maxillary and ethmoid sinuses in CRS patients, using the V4 region of the 16S rRNA gene. Although this region was found earlier to be most informative for short read sequencing (25) and captures the majority of *Bacteria* (26), one limitation of this region is that it cannot capture *Cutibacterium*. This bacterial genus is known to be an important member of the anterior nares (27, 28), so this should be kept in mind in the interpretation of our microbiome results. An overview of the study setup and main findings can be found in the graphical summary in Fig. 6. The microbiome of the anterior nares showed more similarity to that of the sinuses than to that of the nasopharynx. This is unexpected, since the nasopharynx is thought to be a bacterial reservoir for other URT sites due to the mucociliary clearance that moves inhaled particles and bacteria from the anterior nares to the nasopharynx, among other reasons (29). Furthermore, it has been described that several physiological gradients that cause some habitat-specific conditions in the respiratory tract and that consequently can influence the URT microbiome exist along the respiratory tract (30, 31). Our findings confirm previous results from smaller studies with 8 to 19 patients, showing that the microbiome in the nostril and middle meatus could represent the sinus microbiome in CRS patients (15, 32, 33). In a small study setup with 8 participants, where the bacterial topography in the lower respiratory tract was investigated, microbiome continuity between respiratory niches was also suggested, due to the physiological mechanisms of microaspiration (34). The continuity of bacteria in closely related human body habitats is, however, not self-evident, as was already shown, for instance, in the oral cavity, where even closely related niches have unique and significantly different microbiome communities (35). The fact that the microbiome of the anterior nares represents the CRS microbiome better is an important observation for clinicians who cannot access the sinuses, unless they do so during surgery.

Altered bacterial diversity is often a hallmark of chronic polymicrobial diseases that are not caused by a specific pathogen, including CRS (8, 11–13). We observed decreased bacterial diversity in the anterior nares and nasopharynx in CRSsNP patients compared to that in healthy controls but not compared to that in CRSwNP patients (Fig. 6). These results confirm recent work where a trend for a decrease in bacterial richness in the middle meatus compared to that in the controls was found for CRSsNP patients but not for CRSwNP patients (33). However, another study showed decreased bacterial diversity in the middle meatus of CRSwNP patients compared to that in healthy controls (14). In a larger study using middle meatus samples, no significant differences in alpha diversity between control subjects and CRS patients were found (20), and these findings were also confirmed by others (12, 36, 37). These discrepancies might be explained by (i) no or inaccurate phenotyping of CRS in CRSwNP and CRSsNP patients, (ii) the difference in diversity was very small and underpowered in some studies, (iii) differences in control samples, and (iv) alpha diversity is perhaps not a good biomarker for CRS. More specifically, care should be taken when drawing conclusions on bacterial diversity based only on relative microbiome profiling because the difference can be very small and, as such, might be not good enough to predict disease status. Additionally, supplementation with quantitative microbiome profiling approaches might provide additional insights into the role of bacterial diversity in URT health and disease (38). However, optimization of this quantitative profiling is needed, since protocols for high-biomass niches, such as the gut, cannot easily be implemented for low-biomass niches, such as the URT.

Another strength of this study was the comparison of the bacterial profiles from both study groups based on their presence or absence, combined with their relative abundances, to identify indicator species (Fig. 6). The most interesting ASV that was more prevalent and that showed a higher relative abundance in healthy controls was Dolosigranulum pigrum. Previous studies on the URT microbiome in children have investigated the potential protective effects of *Dolosigranulum* for respiratory health (39, 40). *Dolosigranulum* is a member of the lactic acid bacteria (41), which are generally known to be beneficial in the human gut (42) and vagina (43). Future studies are needed to validate the health-promoting effects and industrial application potential of *Dolosigranulum*.

Additionally, several taxa were identified to be possible CRS pathobionts, based on their increased occurrence or relative abundance. We observed an increased relative abundance of C. tuberculostearicum in CRS patients compared to the controls. These findings build further on previous studies reporting an increase in the relative abundance of C. tuberculostearicum in CRS patients (6, 9). Another study revealed that Corynebacterium accolens, which is closely related to C. tuberculostearicum, was the most abundant species in CRS patients but not in the controls (13). In CRS patients, an increase in the relative abundance of S. aureus was measured in nasal polyp tissue (44) and was found to drive Th2-type inflammation (45, 46). In line with the findings presented in literature, our results show that two *Staphylococcus* ASVs were more present and more abundant in the anterior nares of CRS patients than in those of the healthy controls (14). However, in this study the V4 region of the 16S rRNA gene was used, but this could not be used to discriminate between different *Staphylococcus* species, so we could not confirm if these ASVs were S. aureus. Also, two *Haemophilus* ASVs, classified as H. influenzae and H. aegyptius, were more abundant in CRS patients than in healthy controls. Haemophilus influenzae has already been linked with CRS in both culture-based (47) and culture-independent (14, 48) studies. Additionally, in other inflammatory airway diseases, such as severe bronchitis in children, *Haemophilus* has been described as a pathobiont (49). Finally, one *Prevotella* ASV was more prevalent in the nasopharynx of CRS patients than in that of the healthy controls. This genus has been shown to be among the most abundant species in the sinuses of CRS patients, but its possible contribution to the disease etiology remains unknown (10, 50). The exact role of these pathobionts remains to be further substantiated in follow-up work. Notwithstanding these unknowns, the less abundant ASVs should also not be ignored, since they might have an impact as well on interspecies relations in the URT.

Lastly, we explored the association between several patient characteristics and phenotype- and endotype-related variables and specific microbiome features. In our patient group, age, sex, and a history of FESS showed a minor association with the overall microbiome structure. While these associations were statistically significant, their biological significance may be debated, since the effect sizes observed were very small (<2%). This is similar to the findings of gut microbiome studies, where individual predictors of microbial community composition have effect sizes that seldom exceed 4% (51). Surprisingly, we did not find an association between nasal polyps and the overall microbiome structure or the specific microbiome clusters. Also, no associations were found for allergy, asthma, infection, and the inflammatory markers. This is in contrast to the findings of previous studies demonstrating significant associations between the microbiome for asthma and purulence (11). Although our larger multicenter study cohort had the advantage of more statistical power, there were some limitations to our study. More specifically, for some variables, such as medical treatment and a history of smoking, we could rely only on data self-reported by the participants. For instance, the data for previous antibiotic use were based on the question of whether antibiotics were taken in the last 3 months prior to surgery. This might explain why we did not observe differences in microbiome profiles with antibiotic use, while other studies already found a significant impact of antibiotic use on microbiome depletion in different human body sites (52–54). Future studies should pay attention to antibiotic use and monitor the exact timing, type, and dose of antibiotics used before and during surgery.

In conclusion, the microbiome of the anterior nares in patients with CRS was more similar to that in the sinuses than to that in the nasopharynx, indicating that the anterior nares are an important habitat for potential sinus pathobionts. This relevant finding might have implications for the diagnosis, prevention, and treatment of CRS and emphasizes the potential of personalized medical treatment based on the sinus microbiome composition via sampling of the anterior nares. A decrease in bacterial diversity was observed in CRSsNP patients and not in CRSwNP patients, highlighting the difference in pathophysiology between these two phenotypes. Additionally, changes in bacterial diversity probably contribute to disease development more in CRSsNP patients than in CRSwNP patients, or, the other way around, specific conditions in CRSsNP patients might have a larger impact on bacterial diversity. Moreover, certain bacterial taxa, such as C. tuberculostearicum, H. influenzae/H. aegyptius, and one *Staphylococcus* ASV, were confirmed or newly revealed to be potential pathobionts in CRS. Additionally, the association of D. pigrum with the healthy URT, based on its prevalence and relative abundance, provides a first indication that this strain could have potential as a beneficial bacterium in the URT. Future research should focus on mechanistic studies to explore the role and activity of these bacterial taxa in the pathogenesis of CRS and the microbial ecology and stability of the URT.

## MATERIALS AND METHODS

### Study population and sample collection.

One hundred healthy participants were recruited as described previously (24). Briefly, participants between 18 and 65 years old were recruited under study B300201524257 after providing written consent at the University of Antwerp and Antwerp University Hospital during 2015 and 2016 (the study was registered on 23 March 2015 with the approval of the Ethical Committee of Antwerp University Hospital and has been registered at ClinicalTrials.gov under identifier NCT02933983), Participants who had received antibiotics (self-reported) in the previous year or who suffered from acute or chronic airway infections were excluded from the study. Samples were collected from the anterior nares and nasopharynx in a standardized way by the responsible ear, nose, and throat (ENT) specialist with flocked swabs (503CS01; Copan). Patients with CRS (*n* = 225) between the ages of 18 and 65 years that underwent a bilateral functional endoscopic sinus surgery (FESS) were recruited at the University Hospitals of Antwerp and Leuven under the same study (study B300201524257) between July 2015 and June 2018. A diagnosis of CRS was made according to the European position paper on rhinosinusitis and nasal polyps (1). Nasal swab (catalog number 503CS01; Copan) specimens were collected from the anterior nasal cavity and nasopharynx. During FESS, samples from the maxillary and ethmoid sinus were collected. Patients with ciliary dyskinesia, inverted papilloma, or aspirin intolerance were excluded. Written informed consent was obtained from all participants, as was a blood sample, to measure inflammatory markers, and a questionnaire with information regarding the patient’s characteristics and phenotypes was administered (Table 1). Bacterial DNA was isolated from the swabs as described previously (24). In addition, negative extraction controls were extracted at regular time points throughout the study. All samples were obtained within the same study and collected and processed by the same ENT specialists and researchers according to standardized protocols.

### Illumina 16S rRNA amplicon sequencing and quality control of reads, taxa, and samples.

Samples were processed and sequenced as described earlier (24). Briefly, dual-index paired-end sequencing of the V4 region of the 16S rRNA gene was performed on a MiSeq desktop sequencer (catalog number M00984; Illumina). Processing and quality control of the reads were performed for each run separately using the R package DADA2, version 1.6.0 (55). Briefly, this entailed quality filtering of the reads, dereplication, denoising, removal of chimeras, and read classification (see Text S1 in the supplemental material for details). In addition, species with identical V4 sequences were identified for each ASV. The EzBioCloud 16S rRNA gene database (21), downloaded on 8 January 2018, was used as the reference database. The result of these steps was an ASV table with read counts for all ASVs in all samples. After quality control, ASVs not classified to the kingdom *Bacteria* or classified as chloroplasts or mitochondria and ASVs identified as contamination were removed. The concentration of qualitative DNA in each sample was estimated by dividing the number of reads (counted after read and ASV quality control) by the volume of sample pooled for the sequencing run. Samples with DNA concentrations in the range of the negative controls (i.e., negative extraction controls and negative PCR controls) were removed.

10.1128/mSphere.00532-19.1 TEXT S1 Supplemental materials and methods. Download Text S1, DOCX file, 0.03 MB. Copyright © 2019 De Boeck et al. 2019 De Boeck et al. This content is distributed under the terms of the Creative Commons Attribution 4.0 International license.

### Data and statistical analysis.

All data handling and visualization were performed in R, version 3.4.4 (R Core Team, 2018), using the tidyverse set of packages (56) and the in-house package tidyamplicons (github.com/Swittouck/tidyamplicons). All analyses were performed at the ASV level, with the exception of the visualization of the 11 most abundant genera (Fig. 1A) and the clustering of subjects into microbiome types (Fig. 5). Alpha diversity measures, i.e., richness and the inverse Simpson index, were compared using Welch *t* tests with the Holm-Bonferroni correction for multiple testing, where richness was defined as the number of ASVs present and the inverse Simpson index was defined as the inverse probability that two random reads belong to the same taxon. For all beta diversity analyses, the Bray-Curtis dissimilarity was used. Associations between sample covariates and the microbiome were tested using adonis tests (the function adonis of the vegan R package [57]). The differential presence of ASVs between conditions was tested using Fisher exact tests on contingency tables between the variables present/absent and condition (CRS/control). The differential abundance of ASVs was tested using unpaired Welch *t* tests on the relative abundance vectors between the conditions. A rarefied version of the data (1,000 reads per sample) was used for all alpha diversity analyses and for the differential presence analysis. The read depth distribution per sample can be found in Fig. S4.

10.1128/mSphere.00532-19.4 FIG S3 Hierarchical clustering at the genus level for nasopharynx samples. Patient samples were clustered into microbiome clusters based on the abundance of ASVs. Clusters with less than five participants were not used for further analysis. At the end, six clusters remained: *Haemophilus* (cluster 1), *Moraxella* (cluster 2), *Corynebacterium*/*Staphylococcus* (cluster 3), *Streptococcus* (cluster 4), *Staphylococcus* (cluster 5), and *Prevotella* (cluster 7). Download FIG S3, TIF file, 1.1 MB. Copyright © 2019 De Boeck et al. 2019 De Boeck et al. This content is distributed under the terms of the Creative Commons Attribution 4.0 International license.

10.1128/mSphere.00532-19.5 FIG S4 Read depth distribution per sample. Download FIG S4, TIF file, 0.2 MB. Copyright © 2019 De Boeck et al. 2019 De Boeck et al. This content is distributed under the terms of the Creative Commons Attribution 4.0 International license.

### Measurement of inflammatory cytokines in serum of healthy controls and CRS patients.

Serum was collected and stored at −20°C until subsequent analysis. Periostin was measured using a sandwich enzyme-linked immunosorbent assay according to the manufacturer’s protocol (Thermo Fisher, CA, USA). The cytokines IL-4, IL-5, IL-13, and IFN-γ were measured using a multiplex 96-well plate-based assay (MesoScale Discovery, Gaithersburg, MD, USA). A detailed description of the procedure can be found in Text S1.

### Data availability.

The sequencing data were deposited in ENA under accession number PRJEB30316.
