# Supplementary material for: Anterior Nares Diversity and Pathobionts Represent Sinus Microbiome in Chronic Rhinosinusitis
Source: mSphere. 2019 Nov 27;4(6):e00532-19. doi: 10.1128/mSphere.00532-19 (PMC6881717; doi:10.1128/mSphere.00532-19)
Supplement: TEXT S1 [file mSphere.00532-19-s0001.docx]

**Supplementary Materials and Methods**

**Study population and sample collection**

Samples were obtained from healthy participants (n = 100) at the University of Antwerp and Antwerp University Hospital during 2015 and 2016, as previously described (ethical committee approval number B300201524257) (1). Samples from CRS patients (n = 225) were collected in a standardized way by the responsible ENT specialist during functional endoscopic sinus surgery (FESS) at the Antwerp University Hospital and the University Hospital of Leuven between July 2015 and June 2018 (study B300201524257). Diagnosis of CRS was made according to the European Position Paper on Rhinosinusitis and Nasal Polyps (2). Nasal swabs (Copan, 503CS01) were collected from the anterior nasal cavity (indicated as ‘anterior nares’ in the remainder of the manuscript) and nasopharynx. For nasopharynx samples, a sterile nasal speculum was used to minimize contamination with the anterior nares. During FESS, additional samples from the maxillary and ethmoid sinus from CRS patients were collected using a forceps. CRS patients between the age of 18 and 65 that underwent a bilateral FESS were included. Patients with ciliary dyskinesia, inverted papilloma or aspirin intolerance were excluded from the study. A written informed consent was obtained from all participants. Several general and clinical patient characteristics were recorded via a questionnaire and if available checked in the patient’s medical record, including age, gender, Sino-Nasal Outcome Test (SNOT)-22 scores, Visual Analog Scale (VAS)-scores (total symptom score), medical treatment (nasal/oral steroids and antibiotics in last three months), smoking behaviour and history of FESS. Also phenotypic characteristics of self-reported asthma, allergies (based on total IgE and skin prick tests to common inhalant allergens) and nasal polyps were documented. Lastly, different inflammatory cytokines (periostin, Interleukin (IL)-4, IL-13, IL-5 and interferon-gamma or IFN-γ) were determined in the serum samples. Patient information can be found in Table 1. Bacterial DNA from the swabs was isolated as described previously (1).

**Illumina MiSeq V4 *16S* *rRNA* amplicon sequencing and quality control of reads, taxa and samples**

Samples were processed and sequenced as earlier described (1). Briefly, dual-index paired-end sequencing was performed on the V4 region of the *16S rRNA* gene on the MiSeq Desktop sequencer (M00984, Illumina) at the Centre of Medical Genetics, University of Antwerp, Belgium. Samples were spread over six different sequencing runs.

Because of a large difference in quality between the forward and reverse reads, only the forward reads were retained for analysis. This was possible without much loss of information because of the almost full overlap between the forward and reverse reads (251 bp each) in the V4 region of the *16S rRNA* gene (254 bp). The processing and quality control of the forward reads were performed for each of the six runs separately using the R package DADA2, version 1.6.0 (3). All reads containing more than two expected errors were removed. Next, the parameters of the DADA error model were learned from a random subset of one million reads. The reads were then dereplicated, and the quality profiles of identical reads were averaged per base position. This run-specific error model was used to denoise the unique sequences, using the DADA algorithm. This allowed us to infer the amplicon sequence variants (ASVs) to describe the bacteria taxa present. In the next step, the function “removeBimeraDenovo” was run on the denoised reads (or ASVs) to detect and remove chimeric sequences. Finally, reads (or ASVs) were classified from the kingdom to the genus level using the RDP algorithm (4). In addition, species with identical V4 sequences were identified for each ASV. The EzBioCloud 16S rRNA gene database (5), downloaded on 08/01/2018, was used as reference database. The result of these various steps was an ASV table with read counts of all ASVs in all samples.

In the next phase, some ASVs and samples were removed as a result of quality control. ASVs not classified to the kingdom Bacteria were removed, as well as ASVs classified as chloroplasts or mitochondria and ASVs identified as contamination. Next, contaminants were identified by manual inspection of the sequenced DNA extraction kit negative controls and PCR negative controls. The following genera were identified as contaminants and removed from the data: *Acinetobacter*, *Aliivibrio*, *Alloprevotella*, *Citrobacter*, *Enhydrobacter*, *Enterobacter*, *Granulicatella*, *Halomonas, Leuconostoc*, *Marinomonas, Methylobacterium*, *Shewanella* and *Treponema*. In addition, one specific ASV that was unclassified at the genus level but classified to the family Comamonadaceae was also removed. Finally, the concentration of “qualitative” DNA in each sample was estimated by dividing the number of reads (counted after read and ASV quality control) by the volume of sample pooled on the sequencing run. Samples with DNA concentrations in the range of the negative controls were removed.

**Data and statistical analysis**

All data handling and visualization was performed in R version 3.4.4 (R Core Team, 2018) using the tidyverse set of packages (6) and the in-house package tidyamplicons (github.com/Swittouck/tidyamplicons). A unique name was given to each ASV by taking the name of the most specific rank available and adding a number to differentiate it from ASVs with identical taxonomic annotation. The name of the relatively abundant genus CP009312_g was changed to *Lawsonella* (7). A rarefied version of the dataset was created by randomly subsampling 1000 reads from each sample and removing samples with less than 1000 reads. All visualizations and analyses were performed at the ASV level on the unrarefied data except when stated otherwise.

For the visualization of the top eleven genera in a bar chart, taxa were aggregated at genus level. Pairwise comparisons of alpha diversity between sampling locations were performed on the rarefied data using unpaired Welch t-tests. Both richness, a measure for the total number of taxa, and inverse Simpson index, taking number and evenness into account, were calculated. The Holm-Bonferroni method was used to correct p-values for multiple testing. Bray-Curtis similarities were calculated by calculating the Bray-Curtis dissimilarities on the relative abundance data using the vegan package (8) and then subtracting them from 1. The adonis test, to assess the effect of sampling location and participant, was executed using the function “adonis” from the vegan package. Briefly, the adonis function executes a PERMANOVA analysis: a pseudo F-ratio is calculated by comparing the sum of squared dissimilarities within groups with the sum of squared dissimilarities between groups. Statistical significance is assessed by permuting group membership between the observations. All adonis tests were performed using a model with a single predictor, except when assessing the effect of sampling location on the microbiome, in which case the participant was included in the model as a covariate.

The comparison of alpha diversity measures between healthy participants, CRSwNP and CRSsNP was performed in the same way as the alpha diversity comparisons between sampling locations. A general association between the microbiome profiles and health/CRS status was tested using an adonis test. Differential presence plots were generated by counting, for each ASV, the number of control samples (CON) and the number of CRS samples where the ASV was present. For differential presence testing, a contingency table with the variables present/absent and CON/CRS was made for each ASV. Association between those two variables was then tested with a fisher exact test. Differential abundance plots were made by calculating the mean relative abundance of each taxon across all CON samples and across all CRS samples. For differential abundance testing, a Welch t-test for each taxon was performed on the CON and CRS abundance vectors.

To assess the effects of participant metadata (i.e. patient characteristics, phenotypes and inflammatory markers) on their nasopharynx microbiome, an adonis test was performed for each predictor. For each predictor, an association between that predictor and the microbiome was tested separately on all CRS, CRSsNP and CRSwNP samples (samples with missing data were excluded). To visualize the associations with the metadata, the samples were clustered into microbiome types using hierarchical clustering (average linkage) on the Bray-Curtis dissimilarities, calculated on the genus-level abundance data. Next, the associations between clusters and numerical predictors were visualized using boxplots and the associations with the categorical predictors were visualized using mosaic plots.

For further details, see <https://github.com/SWittouck/urt_crs>. Additional R packages that were used are ggrepel (9) to visualize ASV labels on scatterplots, ggpubr (10) to put significance levels on boxplots and ggmosaic (11) for the mosaic plots (12)(12).

**Measurement of inflammatory cytokines in serum of healthy controls and patients with CRS**

Serum was collected from patients with CRS and stored at -20°C until subsequent analysis. Periostin was measured using sandwich ELISA, following manufacturer’s protocol (Thermofisher, California, USA). The cytokines IL-4, IL-5, IL-13 and IFN-γ were measured by incubating serum on a multiplex 96-well plate-based assay that contained antibodies for IL-4, IL-5, IL-13 and IFN-γ. The Meso Scale Discovery plex assays (MesoScale Discovery, Gaithersburg, MD, USA) were performed following manufacturing protocol. Briefly, standard curves were prepared in the supplied assay diluent for human serum, with a range of 40000 to 1.2 pg/ml, depending on the cytokine. Arrays were pre-incubated with 25µl per well of assay diluent for 1 hour. After the pre-incubation, 25µl of sample was added to the appropriate well. The array was then incubated at 4°C overnight. The array was washed with PBS plus 0.05% Tween 20 and 25 µl detection antibody reagent was added. After 1 hour of incubation at room temperature while shaking, the array was washed and the detection buffer was added. Results were read with a Meso QuickPlex SQ120.

1. De Boeck I, Wittouck S, Wuyts S, Oerlemans EFM, van den Broek MFL, Vandenheuvel D, Vanderveken O, Lebeer S. 2017. Comparing the Healthy Nose and Nasopharynx Microbiota Reveals Continuity As Well As Niche-Specificity. Front Microbiol 8:2372.

2. Fokkens WJ, Lund VJ, Mullol J. 2012. European Position Paper on Rhinosinusitis and Nasal Polyps. Rhinology 50:1–298.

3. Callahan BJ, Mcmurdie PJ, Rosen MJ, Han AW, Johnson AJ, Holmes SP. 2016. DADA2: High-resolution sample inference from Illumina amplicon data. Nat Methods 13:581.

4. Wang Q, Garrity GM, Tiedje JM, Cole JR. 2007. Naïve Bayesian classifier for rapid assignment of rRNA sequences into the new bacterial taxonomy. Appl Environ Microbiol.

5. Yoon SH, Ha SM, Kwon S, Lim J, Kim Y, Seo H, Chun J. 2017. Introducing EzBioCloud: A taxonomically united database of 16S rRNA gene sequences and whole-genome assemblies. Int J Syst Evol Microbiol.

6. Wickham H. 2016. tidyverse: Easily Install and Load “Tidyverse” Packages.R package version 1.0.0.

7. Bell ME, Bernard KA, Harrington SM, Patel NB, Tucker T-A, Metcalfe MG, McQuiston JR. 2016. Lawsonella clevelandensis gen. nov., sp. nov., a new member of the suborder Corynebacterineae isolated from human abscesses. Int J Syst Evol Microbiol 66:2929–2935.

8. Oksanen J, Blanchet FG, Kindt R, Legendre P, Minchin PR, O’hara RB, Simpson GL, Solymos P, Stevens MHH, Wagner H, Oksanen MJ. 2018. Vegan: community ecology package. R Packag Version 2 4-6.

9. Slowikowski K. 2018. ggrepel: Automatically Position Non-Overlapping Text Labels with “ggplot2.” R package version 0.8.0.

10. Kassambara A. ggpubr: “ggplot2” Based Publication Ready Plots. R package version 0.1.8.

11. Haley Jeppson HH and DC. ggmosaic: Mosaic Plots in the “ggplot2” Framework. . R package version 0.2.0.

12. McMurdie PJ, Holmes S. 2013. Phyloseq: An R Package for Reproducible Interactive Analysis and Graphics of Microbiome Census Data. PLoS One.
